# A Word of Caution to Hospital Buyers

**Published:** 1916-03-11

**Authors:** 


### A WORD OF CAUTION TO HOSPITAL BUYERS.

### Have Drugs Passed their Highest Price?

It seems desirable to utter a word of caution to those who are responsible for purchasing the supplies of drugs for voluntary hospitals. The constant, and at times rapid, advances in the prices of drugs which were experienced from the very beginning of the war right up to the end of last year, have no doubt made buyers somewhat nervous, and one can well believe that there have been instances in which purchasers have been tempted by quotations slightly below those ruling in the open market to take in stocks long ahead of their immediate requirements. So far such transactions have been shown by market developments to have been fortunate, but it is this very circumstance which may be the undoing of those who think that this experience is likely to be repeated so long as the war lasts. For all that anyone certainly knows to the contrary it is, of course, possible that the upward tendency of prices may continue until the natural law of supply and demand again becomes the governing factor in our markets, but it would be unwise to assume that this will be so.

Since the beginning of this year it is noteworthy that the values of most of those drugs which had risen to such phenomenal figures have been almost stationary, while in several cases the high level of prices which had been reached is by no means firmly maintained. This fact seems to suggest that market conditions have undergone a change of some sort, and consequently it behoves buyers to take particular care to obtain all the information possible concerning the market for any particular drug before committing themselves to large purchases. In the first place, let us examine the market condition of some of the synthetic drugs which before the war had been obtained almost exclusively from Germany. Aspirin now costs twenty-five times the price at which acetylsalicylic acid was formerly obtainable; phenacetin, salicylic acid, and salicylate of soda are each about twenty times the pre-war prices; and, in fact, all the drugs of this class are quoted at what may literally be called famine figures, since the available supplies are far short of the prevailing demand. There is a point, however, at which the demand for an article begins to fall off, and the question arises whether the prices of synthetic drugs have reached this point, and the answer is undoubtedly in the affirmative. Some of them are being replaced in medical practice by old-fashioned drugs which were prescribed before these newer products came into vogue, while a proportion of that large section of the public which doctors itself is finding the habit too expensive to be continued in war-time. And while the demand is steadily declining, the available output is steadily growing, and whereas before the war synthetic drugs were not produced outside Germany except in inconsiderable quantities, the foundations of the industry have now been laid not only in this country but in America, and to-day aspirin, phenacetin, salicylic acid, and sodium salicylate are being produced in commercial quantities in factories in and near London, and are also being imported from the United States. The available output does not, of course, approach the volume of Germany's output in normal times, but it must be remembered that during the earlier period of the war all this country had to depend upon were unexhausted stocks and the small supplies that dribbled in from neutral countries. Although the scarcity of available raw materials precludes the probability of any great reduction in the prices of the finished products, the fact that they are being produced here tends to put a brake on the advancing tendency.

Turning to the vegetable drugs, it seems clear that those for the supply of which we were formerly dependent mainly upon Central Europe must become scarcer and dearer as the war drags on. Take, for instance, belladonna root; this is so extremely difficult to obtain that extraordinary figures are asked for the small lots available; while some idea of the scarcity can be obtained from the fact that instead of being sold by the hundredweight it is now quoted at so much per pound. On the other hand, such vegetable drugs as come from British Dominions, from America, and other places beyond the seas will probably continue to be governed by ordinary market conditions; and, except for the increased cost of labour and freight, there seems to be no reason to expect any remarkable upward movements in prices save such as are due to short crops or larger demands.

A product which is causing buyers some anxiety at the moment is cod-liver oil, the price of which has advanced considerably since the beginning of the Norwegian cod-fishing season a few weeks ago. In this case it is difficult to know what to advise. Judging from the quality of the catch secured at the opening of the season, at least a good average yield, and consequently average prices, might have been expected had the times been normal. Under existing circumstances, however, it is impossible to base an opinion on any data at present available; for though the cod may be large and plentiful the fishermen are few, since they are able to obtain higher wages on ocean-going steamers. No one can foresee what the ultimate yield of oil will be, and even if one could, it would be no reliable guide to prices, as Germany may again be a large purchaser. Another question that is no doubt exercising the minds of some hospital buyers is whether it would be prudent to replenish their stocks of bromides at the present prices. This again is a difficult question to answer, but at the moment the tone of the market is not quite so firm as it was a short time ago, and it might not be altogether unwise to wait a little before coming to a decision. These remarks, although they apply specifically to a few only of the more important and costly drugs, will suffice to show that it is now more necessary than ever that buyers should exercise great caution in making purchases.

